# Advances in the Study of Bacterial Toxins, Their Roles and Mechanisms in Pathogenesis

**DOI:** 10.21315/mjms2022.29.1.2

**Published:** 2022-02-23

**Authors:** Ciamak Ghazaei

**Affiliations:** Department of Microbiology, University of Mohaghegh Ardabili, Ardabil, Iran

**Keywords:** bacteria, toxin, pathogenesis, mechanisms

## Abstract

Pathogenic bacteria use various mechanisms to cause infections in the host. Bacterial pathogens express a wide range of molecules. Bacterial toxins have a poisonous substance created and secreted at the extracellular or intracellular level, making these bacteria pathogenic. Two common types of toxins, including exotoxins and endotoxins, have numerous modes of action. Toxin-antitoxin (TA) modules act on their bacterial cells during stressed conditions and help their remaining population survive. Cytolethal distending toxins (CDTs) are genetic, modulating the eukaryotic cell cycle and targeting the immune system of the host. This review discusses toxins and recent discoveries about the mechanisms involved in the pathogenesis of the TA system and CDTs in pathogenic bacteria.

## Introduction

There are two mechanisms by which bacteria can cause a disease condition: i) invasion and inflammation, and ii) toxin production (1). Bacterial toxins are toxic elements, inflicting pathogenic characteristics to some produced microbes. Depending on the toxins type, concentration and affected cell, their consequences may range from a single cell to tissue or organ failure, the innate and adaptive immune system manipulation, and the nervous system impairment. A genetically controlled capability of some microbes to produce toxins is called ‘toxigenicity,’ which causes adverse effects leading to a variety of diseases referred to as ‘toxinoses.’

These toxins are transported by the blood or lymph, leading to various symptoms of diseases, i.e. fever, diarrhoea, shock and cardiovascular disturbance. Some toxins cause the inhibition of protein synthesis, destruction of blood cells and vessels, and disruption of the nervous system leading to spasms. When toxins are present in the blood, it is ‘termetoxemia.’ The term ‘toxin’ is called ‘virulence factor,’ as a molecular component released by the bacteria that interfere with the immune system’s mechanisms to promote colonisation harmful to the host. Based on the position, there are two general types of toxins, including exotoxins and endotoxins (2–5).

## Types of Exotoxins

Exotoxins are classified into three types, based on their structures and functions:

- superantigens
- membrane disrupting toxins
- A-B toxins

### Superantigens (Type I Toxins)

Superantigens are classified as type I toxins as they do not enter the cell. This provokes immune responses, such as a shock induced by lipopolysaccharides (LPS) or lipoteichoic acid (LTA). However, bacterial protein toxin that causes shock syndrome is referred to as toxic shock syndrome toxin (TSST), differing from LPS or LTA.

TSST forces unnatural associations between macrophages and T cells, causing an outflow of cytokines to trigger a shock response due to superantigens. Synergistic action of superantigens with LPS increases the ability of LPS to stimulate cytokine release. These toxins are impacted by binding to the major histocompatibility complex class II (MHC II) of macrophages and the receptors on T helper cells (T_H_ cells). Antigen-presenting cells (APCs), such as macrophages, cleave antigens into peptides and display with MHC II on macrophages surface called ‘MHC-peptide complex’; only a few T_H_ cells have receptors to recognise this complex. These non-specific binding of bacterial toxins, i.e. superantigen and MHC II on macrophages, leads to the formation of more numbers of macrophage-T_H_ cell pairs. When macrophages stimulate such T_H_ cells, they release cytokine, including interleukin-2 (IL-2), as an immune response, causing high levels in the bloodstream. The rise in levels of IL-2 in the bloodstream causes symptoms, such as nausea, vomiting, malaise, diarrhoea, fever, shock and even death (3, 4).

### Membrane Disrupting Toxins (Type II Toxins)

Membrane disrupting toxins disrupt the plasma membranes of the host cells and cause cell lyses. Such toxins have two different roles in disrupting the plasma membrane of host cells.

In some cases, their primary role is to kill the host’s immune cells, such as phagocytes. In other cases, bacteria use phagocytes to escape from phagosomes and enter the cell’s cytoplasm before phagolysosomal fusion. There are two types of membrane disrupting toxins. One type forms protein channels in the plasma membrane. The osmotic strength of the host cell cytoplasm is higher than that of the surrounding environment causing pore formation in the membrane, leading to swelling of the cell. As the membrane is not strong enough for this sudden fluid influx, it causes cell lyses.

The second type is an enzyme that disrupts the integrity of membrane phospholipids. Enzymes, such as phospholipases, cytolysins or hemolysins, have similar actions to destroy the integrity of cell membrane lipids. Charged group on heads of lipid bilayer stabilises host cell membrane, hence removing this head group by phospholipases; membrane structure gets destabilised causing host cell lyses. Other types of phospholipases cleave at other sites with the same effect to destabilise the host cell membrane.

Membrane disrupting toxins are often called ‘heamolysins’ (by staphylococci and streptococci) because red blood cells use such type of toxin system. However, cell-membrane is the main target of such toxins; hence, they can act on the other cell types termed ‘cytolysins.’ Some toxins can kill phagocytic leukocytes (white blood cells) by forming protein channels called ‘leukocidins’ (mostly by staphylococci and streptococci). Leukocidins can act against macrophages and the phagocytes present in tissues.

### A-B Toxins (Type III Toxins)

The first studied toxins are named A-B toxins designated as A and B, based on two polypeptide chains.

A-subunit is an active enzyme with toxic activity, and B-subunit is the binding component, which can bind exotoxin to the specific receptors on the human cell membrane, e.g. diphtheria, tetanus, botulism, and cholera toxins and the enterotoxin of *Eschericia coli* (*E. coli*).

A-part (enzymatic component) catalyses the reaction of adenosine-diphosphate (ADP)-ribosylation, i.e. the addition of ADP-ribose to the target protein in the human cell. This ADP-ribosylation causes inactivation or hyperactivation of target protein-inducing diseases. The secretion system of bacteria releases exotoxins and transports them into extracellular space, while others release them into the mammalian cell.

Direct transport of exotoxins into mammalian cells is effective because it has less or no exposure to the host’s immune system or antibodies in the extracellular space. There are six types of secretion systems, of which the type III secretion system (also called ‘injectosome’) is the most important in virulence. *Pseudomonas aeruginosa*, strains with this type of secretion system, are more virulent than those without. Some other Gram-negative bacteria, such as *Shigella* sp., *Salmonella* sp., *E. coli* and *Yersinia pestis*, utilise injectosome.

## Endotoxins Within Bacterial Cell, Released After Its Death

They are present in the outer portion of the cell wall of Gram-negative bacteria. This outer membrane surrounds the peptidoglycan layer of the cell wall and consists of LPS and phospholipids. The lipid portion of LPS, called lipid A, acts as an endotoxin. Most endotoxins are lipids, while exotoxins are proteins (3, 4). Major sites of action for endotoxins are macrophages. Bacterial lysis in macrophages causes a release of these endotoxins (LPS) from the surface of Gram-negative bacteria in the form of small pieces, which bind to LPS-binding protein in the plasma.

The activation of a signal cascade in macrophages results in the synthesis of interleukin-1 (IL-1), tumour necrosis factor (TNF) and nitric oxide. Fever and hypotension are the main silent features of septic shock, as well as tachycardia, tachypnea and leukocytosis. Septic shock can cause deaths in patients with an almost 30%–50% mortality rate.

Along with endotoxins, some surface molecules of Gram-positive bacteria are also responsible for septic shock. Lipid A is the toxic component that contains fatty acids, such as β-hydroxy myristic acid, while other fatty acids differ in species. Polysaccharide core is present in the middle of a molecule with similar chemical composition in members of the same genus and protrudes from the bacterial surface. The somatic antigen (O antigen) present on the exterior surface is a polysaccharide, an important antigen of Gram-negative bacteria with large antigenic diversity.

A fever is a result of the released IL-1 (endogenous progeny) and IL-6 by the macrophages. Interleukins act on the hypothalamic temperature-regulatory centre. Nitric oxide induces vasodilatation, TNF causes an increase in capillary permeability, and Bradykinin can induce vasodilation and an increase in capillary permeability. All these factors are responsible for hypotension, shock and impaired perfusion of essential organs.

Disseminated intravascular coagulation is a result of the activated coagulation cascade, causing thrombosis, a petechial rash and tissue ischaemia, and finally, the failure of vital organs. In hospitals, septic shock is caused by the presence of endotoxins in intravenous fluid, leading to fever in patients (1, 3).

This review discusses two such bacterial toxins: i) toxin-antitoxin (TA) modules (6) and ii) cytolethal distending toxins (CDTs) (1, 3, 7).

### Toxin-Antitoxin Modules — Suicide, the Best Survival Strategy

Kedzierska and Hayes (6) described a toxin as a product of different plants, animals and microorganisms that can inhibit the growth of competing species and help in defense by assisting infection into a host species to provide benefits. Bacteria can yield various bacterial toxins, potent antibiotics able to kill/inhibit other microorganisms not harmful to producers. TA modules are mostly encoded by bacteria and Archaea species. Some bacterial genes synthesise products that inhibit their own cell growth or death during overexpression, including the eukaryotic apoptosis mechanism. TA modules is also taking part in epigenetic regulatory mechanism in bacteria (8, 9, 10). Bacterial TA modules comprise a protein toxin that inhibits cell growth by interfering with vital processes and a protein or RNA antitoxin that protects its own cell by sequestering the toxin’s action, either by directly inhibiting or controlling its production (6, 9, 11). TA modules are different types of systems since both toxin and antitoxin are not secreted but act inside the bacterial cell produced by. The transcription and translation of toxin-antitoxin genes are tightly coupled and encoded in a single operon, ensuring their stoichiometric ratio (12–15).

Toxin proteins are small (< 10 kilo Daltons) and compact, with globular fold with β-sheets in their core. Protein antitoxins lack structural patterns and protein folding, making them vulnerable to protease digestion; the in vivo lifetimes of both toxins and antitoxin impact TA modules action. Toxins have more lifespan than antitoxins; it is degraded by cellular nuclease or proteases (6, 16–18). TA modules have been first reported as a system leading to plasmid maintenance. Further genome sequencing techniques help discover multiple TA modules encoded in the chromosomes (12, 13, 19), which are related to functions linked to distinct metabolic and growth control processes occurring in response to adverse environments (18, 20–21). Chromosomal TA modules induce programmed cell death (PCD) in the bacterial population as an altruistic strategy to survive in adverse situations (22). Bioinformatics analysis provides evidence that many chromosomally has integrated mobile genetic elements (MGE), such as pathogenic islands and conjugative or mobilisable integrons containing TA modules (23, 24), since chromosomal TA modules are similar to plasmid TA modules and can maintain genetic elements in the genome, contributing to MGE-encoded resistance or virulence determinant (20, 25).

Mechanism of ‘abortive infection’ provides innate bacterial immunity during bacteriophage infection through altruistic suicide (26). Also, it has been noticed that ‘bacterial persistence’ by TA modules provides recalcitrance in chronic infections, such as urinary tract infections (uropathogenic *E. coli* in urinary tract infections) and tuberculosis. This recurrence creates significant challenges in the treatment of diseases (8, 10, 27).

### Types of toxin-antitoxin modules

The mechanisms used by antitoxin to inhibit the activity of its associated toxin were classified into six types (10, 2). In all these types of TA systems, toxins are mostly proteins but the natures of antitoxins differ. TA modules of types II, IV, V and VI are protein-natured antitoxins, and types I and III are small regulatory RNAs (21).

### Toxin-antitoxin and their role in bacterial pathogenesis

TA modules are widely distributed in bacteria. Yamaguchi et al. (10) indicate at least 36 TA modules to be in the *E. coli* genome, while more than 80 TA modules are in *Mycobacterium tuberculosis* (*M. tuberculosis*) H37 Rv strains (9, 13, 18, 29). These large numbers of TA modules in pathogens, such as *M. tuberculosis*, are involved in the mechanisms, including bacterial persistence; biofilm formation helps bacteria develop multidrug resistance increasing the pathogenicity of bacterial cells (13). TA modules can be involved in pathogenesis by forming resistant bacterial communities, causing chronic and recurrent infections (2) (Table 1).

### Mycobacterium tuberculosis — toxin-antitoxin modules and bacterial persistence

*M. tuberculosis* causes tuberculosis, pulmonary infection with a high mortality rate (5, 30). Prolonged infections of *M. tuberculosis* are caused due to the ability of bacteria to evade host defense systems (5, 29). TA systems create antibiotic resistance in strains of *M. tuberculosis* (29, 31).

More than 80 TA systems have been found in the *M. tuberculosis* chromosome, mostly seeming to be associated with the persistence phenomena in their pathogenic strains (29). TA modules are stress response elements that slow down microbe’s physiological activities by inhibiting biological processes, including DNA replication, RNA and protein translation. This metabolic shutdown helps the bacterial cell to enter into the persistent state, inflicting tolerance against antibiotics (5, 13). Emerging evidence has revealed that latency and persistence are linked, where TA modules modulate the physiological activity of microbe (6, 32). Nutrient starved culture of *M. bacterium* has shown an increase in its TA proteins, pointing towards the role of TA modules in creating a dormant state in bacteria (5, 33). TA systems are ubiquitous and highly conserved in the members of the *M. tuberculosis* complex (Mtbc), such as *M. bovis*, *M. africanum*, *M. cannetti* and *M. microti*. This Mtbc genome harbours TA modules belonging to *rel*BE, *par*DE, *ccd*AB, *hig*AB, virulence associated protein *(vap)*BC, *Yef*M/*Yoe*B and *maz*EF families, one tripartite type II TAC (toxin-antitoxin-chaperone) system controlled by a *Sec*B-like chaperone, three potentially type IV systems and eight uncharacterised putative TA modules (31, 34).

The bacterium encodes two functional *rel*BE cassettes and one *Yef*M-*Yoe*B module up-regulates it in response to nitrogen starvation and oxidative stress and down-regulates in response to hypoxia, signifying their roles in persistence (5, 13).

Exposure to certain antibiotics triggers increased expressions of the *rel*E and *Yoe*B toxin genes (5, 35). *Rel*E and *Yoe*B toxins are homologs, whereas the *rel*B and *Yef*M antitoxins are unrelated (36). Korch et al. (37) report that during the early or middle stages of infection, i.e. after phagocytosis of *M. tuberculosis* by human macrophages, genes of the *rel*BE and *Yef*M-*Yoe*B are not expressed. However, in the late infection stage, genes encoding one of the two *rel*E toxins, the *Yoe*B toxin and one of the two *rel*B antitoxins are expressed. They explain that in *E.coli rel*BE system ‘delayed-relaxed response’ by synthesising stable RNA molecules (tRNA and rRNA) due to amino acid starvation is observed. *Rel*BE has been shown to promote reversible cell cycle arrest during starvation stress; nevertheless, macrophages play a role in immunity by phagocytosis of infected microbe, the expression of these TA genes at the late stage of infection shows its role in mycobacterium survival. They validate the role of *rel* proteins in *M. tuberculosis* growth regulation and suggest that *rel* proteins are required for the survival within human macrophages (5, 37).

Gupta et al. (38) report that introducing the *Par*E2 gene in the *M. smegmatis* strain help to switch itself non-culturable under oxidative stress, and *par*E toxin inhibits bacterial growth in oxidative stress, helping bacteria to prevail in macrophages and go into the dormant state. He proves that the *Par*DE2_Ms_ system deals with the tolerance and adaptation to the environmental stress in bacteria involved in mycobacterial pathogenesis. TAC_Mt_ (toxin-antitoxin-chaperone) system is regulated by the interactions between chaperone and antitoxin to prevent the degradation of the chaperon, conserving in the Mtbc and showing an increase in their activity in response to DNA damage, heat shock, nutrient starvation, hypoxia, drug-persistence and host phagocytes (5).

*Maz*EF_Mt_ is the second family with the most members in *M. tuberculosis*. Tiwari et al. (39) have studied this TA family; they have shown that it has nine different *maz*EF-like loci, from an active toxin encoded by only three loci (*maz*EF3, *maz*EF6 and *maz*EF9). An extensive study of these TA modules under different stress conditions (oxidative, nitrosative, nutrient-limiting and low-oxygen stresses), which can mimic host’s defense mechanisms that pathogen encounters during infections in humans, reveals that such systems have different expression patterns: i) toxin gene *maz*F9 is expressed during oxidative stress; ii) genes *maz*F6 and *maz*F9 appear during nitrosative stress and iii) all of these three genes are induced when bacteria are exposed to either nutrient starvation or hypoxia. In all these cases, transcripts of toxin genes are more than that of antitoxin genes. Mutants of *M. tuberculosis*, without *maz*F3, *maz*F6 and *maz*F9 toxins, show lower survival compared to wild-type strains, proving their role in infections in the in vivo model of guinea pigs. In Mtbc, *maz*F homologs have different substrates specificities; for instance, Rv1991c (*maz*F6), Rv2801c (*maz*F9) and Rv1495 (*maz*F4) cleave single-stranded RNA at U↓CCUU, U↓AC and U↓CGCU, respectively. Also, Rv1102c (*maz*F3) cleaves 23S rRNA at the UU↓CCU site contributing to the persistence in *M. smegmatis* (*Msm*) for kanamycin- and gentamycin, proving their role in the protection against certain drugs (39).

*Vap*BC TA family is involved in the addiction system of a virulence plasmid of *Salmonella dublin* (34). Out of 88 known type II TA gene families of *M. tuberculosis*, 45 are of *vap*BC family, making it the most abundant TA gene family in Mtbc. *Vap*C toxins are PIN (pilT amino-terminal) monodomain endoribonucleases, which comprise three or four acidic residues that coordinate with Mg^2+^ ions in their active site.

*Vap*B antitoxins prevent this Mg^2+^ ion binding to the active site of *vap*C toxins; they are related to the families of transcriptional regulators or DNA binding domains associated with *vap*C toxins (34, 40). Lee et al. (41) demonstrate that *vap*C30 involves ribonucleases activity, with its cofactor magnesium to inhibit cellular growth (48). Winther et al. (40) have studied *vap*C20’s possible role in cleavage and cellular arrest, suggesting its role as an endoribonuclease that cleaves between nucleotides G2661 and A2662 in the helix 95 of 23S rRNA (40). These nucleotides are conserved rRNA sequences, the SRL (sarcin-ricin loop). G2661 and A2662 are essential for the function of the SRL loop and involved in elongation factor-TU (EF-Tu) and EF-G-mediated GTP hydrolysis (40–41). During translation, the elongation process of the SRL loop is required for anchoring of EF-G to the ribosome during translocation (42), and *vap*C20 cleaves the SRL loop results in the translation as a potent inhibitor of global translation (40).

### Cytolethal Distending Toxins — Genotoxins

CDTs were identified in some *E. coli* strains, *Shigella* and *Campylobacter* spp. (43–45). After their initial discovery, CDTs were discovered in diverse groups of Gram-negative pathogenic bacteria, where these CDTs target and modulate the eukaryotic cell cycle by inducing DNA lesions. Hence, CDTs are known as genotoxins (4, 45). *E. coli* CDTs (Ec-CDTs) were involved in the arresting cell cycle at the G2/M phase, causing mammalian cell intoxication (46).

Structures of CDTs subunits and their involvement in cell association induce toxicity CDTs as a tripartite toxin, composed of three subunits — CDTA, CDTB and CDTC, which are encoded by the three genes organised in one operon. This A-B_2_ exotoxin has active subunit CDTB and two binding subunits, CDTA and CDTC. The active CDTB subunit is functionally and structurally homologous to mammalian DNase I. CDTA and CDTC bind holotoxin to the target cell’s plasma membrane, and entry of active CDTB subunit then translocated into the nucleus causing DNA lesions (47–50). All CDTs have similar actions; however, their amino acid sequences differ in bacterial species (51, 52). CDTB sequences are conserved in all CDT-producing bacterial species; however, CDTA and CDTC subunits differ (51, 52–55). CDTs can bind specifically to the cholesterol of lymphocytes and macrophages. This cholesterol-binding depends on the amino acid sequences, cholesterol recognition amino acid consensus (CRAC) is encoded within the CDTC subunit. Mutations in CRAC results in reduced toxin binding, internalisation of CDTB subunit and finally affects toxicity induced by CDTs (53, 56–59). McSweeney and Dreyfus (56) observed that some CDTs are not dependent on cholesterol binding and derived from different pathogens possess requirements to intoxicate host cells having different receptors (53, 60–61). Lipid membrane microdomains might be involved in CDT-mediated immune toxicity. With the help of confocal microscopy, they observed that subunits are with GM1-enriched membrane regions, which are characteristics of membrane rafts and further western blot analysis revealed the presence of CDT peptides in isolated lipid rafts (58–59).

## Cytolethal Distending Toxins Act Like Enzymes

### CDTB Acts as DNase

There were 3 ways used to study the association of DNase activity with CDTB subunit:

- demonstration of in vitro DNase activity of CDTB subunit
- nuclear localisation of CDTB and
- activation of DNA damage response (DDR) (53)

Several investigations on CDTB subunit isolated from various bacteria showed denaturation or relaxation of plasmid DNA in vitro (50, 53–54). To translocate, an active CDTB subunit from the cell surface to its nucleus to act on its enzymatic substrate.

When purified, *E. coli* CDTB subunit was introduced by the electroporation method and *Campylobacter jejuni* (*C. jejuni*) CDTB subunit was introduced by microinjection in Cos-7 cells. These studies on different bacteria proved that the CDTB subunit has nuclear localisation signal (NLS) sequences, leading to the nuclear localisation of CDTB subunit (51, 53). Nishikubo et al. (57) and, McSweeney and Dreyfus (56) reported that mutants lacking these NLS sequences showed impaired nuclear localisation, and such mutants fail to intoxicate cells. Fahrer et al. (58) studied the effects of CDTs on mammalian fibroblast and compared them with the effects of ionising radiation. They show that CDTs can induce DNA damage response (DDR), leading to double-stranded breaks in DNA and ionising radiation (IR) induces single-stranded breaks in DNA (58). The DNA damage-related activation of checkpoints involves three stages:

- the recognition of DNA damage by sensor proteins (MRN and Ku complexes, RPA), which rapidly activate specific phosphatidylinositol 3-kinase-related protein kinases (ataxia telangiectasia mutated [ATM], ataxia telangiectasia and Rad3-related [ATR] and DNA-dependent protein kinase [DNA-PK])
- the signal amplification by transducing proteins (CHK1, CHK2)
- activates an appropriate cellular response by effectors proteins (p53, CDC25, etc.). This cell response initiates the cell cycle regulation, the activation of DNA repair pathways and in some cases, cell death pathways. Two key signaling pathways activation happens as a response to DNA damage: the ATM-CHK2 and the ATR-CHK1 pathways (47, 58–59). Guidi et al. (60) revealed that, even if cells survive from CDT intoxication, DNA damage occurred by CDT activity causes genetic instability and may promote carcinogenesis (53, 60).

## CDT Targets Host Defence Systems and Play Role Invirulence

CDT functions as a tri-perditious toxin by targeting host defenses at three levels:

- promotion of infection by modulating epithelial cell growth and survival
- promotion of inflammatory responses by activation of inflammasome leading to cytokine maturation and release and
- impairment of acquired immunity due to inhibiting B- and T-cell proliferation and survival (53). CDTs from microorganisms, regardless of their source, are capable of intoxicating cells or cell lines.

In proliferating cells, intoxication happens by altering cell morphology, by arresting the cell cycle at the G2 phase to inhibit the cell growth and cell death by activating apoptotic cascade (53, 62). Bacteria that produce CDTs, exhibit an affinity to colonise in mucocutaneous tissue such as oral, gastrointestinal, urinary and respiratory tracts cause infections in humans. CDTs can also alter the actin cytoskeleton and disturb focal adhesion and microtubule network leads to a decrease in intestinal epithelial cell adherence (53, 63). Suguimoto et al. (66) studied Aggregatibacter actinomycetemcomitans CDT (i.e. Aa-CDT) and reported that Aa-CDT participates in periodontitis and other non-oral infections. They tested the role of Aa-CDT in disrupting macrophage function by inhibiting phagocytic activity and also affecting the production of cytokines.

They co-cultured murine macrophages with wild-type *A. actinomycetemcomitans* or a CDT^−^ mutant. Generally, macrophages trigger inflammatory reactions in the host due to the presence of pathogens. Wild-type strains have shown a reduction in phagocytic activity compared to CDT^−^ mutants. While recombinant Aa-CDT [Aa(r)CDT] with CDT^−^ mutants have shown that diminished phagocytic is quite similar to suggesting the role of CDT to modulate the nitric oxide production and increase the levels of IL-1b, IL-12 and IL-10. TNF-α did not change in co-culture assays but showed an increase in the presence of Aa(r)CDT, proving the active role of Aa-CDTs to diminish the phagocytic activity and in the modification of inflammatory cytokine balance (64).

Shenker et al. (61) revealed that CDT induces pro-inflammatory cytokine response by macrophages, related to the CDT-mediated activation of caspase-1 and which in turn is dependent upon the activation of NLR family pyrin domain containing 3 (NLRP3) inflammasome.

CDT-treated macrophages induce the production of IL-1β, TNF-α, IL-6 in 5 h and when exposed to CDT for 48 h, they release IL-18 (65). Shenker et al. (61) show CDTB’s role as phosphatidylinositol phosphate (PIP) phosphatase. CDTB mutants lacking lipid phosphatase activity but with their DNase activity intact failed to induce cytokine release supporting the role of phosphatase in the cytokine release. Svensson et al. (68) investigated the effects of *Haemophilus ducrei* CDTs (HdCDTs) on circulating human hematopoietic cells, including T- and B-cells, monocytes and poly-morphonuclear cells (PMN). They showed that: i) HdCDT inhibits mitogen-induced proliferation of circulating human T-cells and ii) it also inhibits the proliferation of B-cells affecting the immunoglobulin production and inhibiting B-cells and T-cells involved in acquired immune response in the host (66).

## *E. coli* CDT and Its Potential Role in Colorectal Cancer

Johnson and Lior (44) reported that Ec-CDT shows its association with gastroenteritis, demonstrating the presence of CDTs in clinical isolates of enteropathogenic *E. coli* (EPEC) (52, 61). Five variants of CDT sequences were detected in EPEC and recently in Shiga-toxin-producing (Stx) *E. coli* (88, 89). Such CDT-positive strains of *E. coli* are found to be involved in uremic syndromes (by uropathogenic *E. coli* [UPEC]) and watery diarrhoea and cause cell cycle arrest to the distend cells and apoptosis mechanisms (54, 67). CDTs have an active role in tumorigenic phenotype because of DNA damage mechanisms. He et al. (72) study on *C. jejuni* proved CDTs active role in colorectal tumorigenesis (68).

Some evidence revealed that human micro-biota are involved in tumorigenesis, mostly in colorectal cancer (CRC); thus, Graillot et al. (71) researched Ec-CDTSs to assess their genotoxic effects on human colorectal cell lines. They proposed Ec-CDTSs might be promoting CRC. Cellular outcomes after acute and chronic exposure to CDT have an active role in promoting malignant transformation in human colon epithelial cells (HCECs). A comparative study was done for isogenic derivatives cell lines of the normal HCECs with those which can mimic the mutations of three significant genes found in CRC genetic models: adenomatous polyposis coli (APC), Ki-ras2 Kirsten rat sarcoma viral oncogene homolog (KRAS) and tumour protein 53 (TP53) (69).

Fearon and Vogelstein (73) explained that mutation-driven multiple pathways in key suppressors and oncogenes’ activation during CRC progression lead healthy tissue to dysplastic adenoma and, finally, carcinoma, in almost 70%–80% CRC cases. One of the earliest genetic changes observed during CRC progression is truncating mutations in APC tumour suppressors (70, 71). This APC plays a vital role in various pathways like cell adhesion and migration, cell cycle control, apoptosis, chromosome segregation, Wnt/b-catenin signaling and recently in DNA repair regulation (72, 73).

Bonnet et al. (76) researched the possible involvement of normal intestinal micro-flora *E. coli* in the cancer risk and increase in numbers of *E. coli* in colon tumours than in colon mucosa. However, pathogenic *E. coli* strains synthesise various virulence factors, such as CDTs, cytotoxic necrotising factor, cycle inhibiting factor and colibactin, which are collectively referred to as ‘cyclomodulins’ (74). Buc et al. (77) study revealed that these cyclomodulins producing B2 *E. coli* (one of the pathogenic strains out of four main phylogenetic groups A, B1, B2 and D) is associated with Crohn’s disease, chronic inflammatory bowel disease and it showed an increased in colon tumour biopsies supports *E. coli* in colon carcinogenesis. They also proposed that this promotion of carcinogenesis is probably not due to bacteria alone, but also the host’s susceptibility plays a pivotal role too along with other factors (75).

## Recent Discoveries: Deciphering the Role of TA Modules and CDTs in the Development of Chronic Diseases

Bacterial toxins protect the bacteria. The activity of toxins is studied to determine various infections occurrence. Bacteria encode toxin-antitoxin modules.

Firstly, they were connected with plasmid maintenance but later found their roles in cellular processes like DNA replication and t-RNA translation in bacteria. There are six different types of TA modules. TA modules show their effects on bacterial cells that produce them instead of the host system in which they reside. TA modules consist of a stable toxin and degradation prone antitoxin (either RNA or protein [enzyme/competitive substrate for the toxin]), which counteracts the action of its toxin by acting as a direct inhibitor or by controlling the production of the toxin. These regulatory mechanisms of TA modules help the bacterial population survive in stressful conditions by bacterial persistence, biofilm formation abortive infection by developing multidrug resistance (6, 9, 14, 21). During World War I, the therapeutic potential of phages was recognised and prepared to treat dysentery in soldiers. This use of bacteriophage as a bactericidal agent was practiced before the discovery of antibiotics led to the process of ‘abortive infection’ found to be involved in protecting bacteria from the bacteriophage infection (mostly in cultures grown in the laboratory), and it also interferes with the use of bacteriophages as therapeutic agents called ‘phage therapy.’ TA modules act as an antiphase system that provides resistance to phage infections.

Various TA model studies also show their important role in downregulating their metabolism and even causing programmed cell deaths in certain parts of their population. Mechanisms such as bacterial persistence and biofilm formation help the rest of the population survive by maintaining the stoichiometric ratio of toxin and antitoxin. TA modules in pathogenic bacteria are involved in developing multidrug resistance and create problems during the treatments of disease. Such infections by pathogenic bacteria always create finding a perfect therapeutic cure. The mechanism of the TA module increases the pathogenicity of microorganisms. *M. tuberculosis* consists of 88 different types of TA modules discovered to date. Research findings of these systems revealed that they are highly involved in pathogenesis by mechanisms like bacterial persistence and by developing multidrug resistance. Dormancy and multidrug resistance make it challenging to develop therapies for tuberculosis infections.

CDTs are genotoxins, target and modulate the eukaryotic cell cycle by inducing DNA lesions. They also target host defense systems by promoting inflammatory responses, triggering activation of inflammasome and cytokine release, and inhibiting B- and T-cells proliferation, leading to impairment of acquired immunity. Hence, CDTs hamper the host immune system and help in pathogenic bacteria infections (61). Bezine et al. (78) reported that during low doses of CDTs toxins, DNA lesions lead to replication-dependent double-strand DNA break (DSB) formation that indicates the possibility of non-DSB repair mechanisms, which might be contributing to the CDTs cell resistance. They confirmed that the two major DSB repair mechanisms, homologous recombination (HR) and non-homologous end joining (NHEJ), manage CDT-induced lesions. They demonstrated that single-strand break repair (SSBR) impairment alerts cells to CDT, but not the nucleotide excision repair. Their study concludes that cells can survive CDT-induced DNA damages with the help of different repair pathways.

Repair mechanisms play an imperative role in resisting CDTs-induced DNA damages and help survive the host’s cells. Hence, studying CDTs actual mechanism, which helps pathogenic bacteria to create infections, and the body’s repair mechanism, which can provide resistance to such effects of CDTs (76). Some Gram-negative bacteria such as *E. coli* as a part of normal microflora in the human gut can produce CDTs. *E. coli* can produce CDTs (Ec-CDTs). In vitro study revealed the role of Ec-CDTs in promoting colorectal cancer in human colonic epithelial cells. Since *E. coli* is a part of normal flora in the human gut, studying the fundamental role of Ec-CDTs in the development of cancer becomes essential (77, 78).

## Conclusion

In pathogenic bacteria, bacterial toxins play a beneficial role in microbe-host interactions, progressing disease conditions in their host system. As discussed earlier, in TA modules of pathogenic bacteria, bacterial toxins influence their system, inducing PCD or slowing down the metabolism in its population. They help bacteria downplay in the host’s system, aiding the survival of a few bacterial cells in the population. Hence this programmed cell death by the bacterial TA modules acts as a best-programmed cell survival strategy in the pathogenic bacteria; during stressed conditions. While CDTs cause damage to the cells of a host by modulating eukaryotic cell cycles, they can also target the host immune system and cells, thereby helping pathogenic bacteria to survive in the host system. Some investigations informed about the involvement of CDTs in carcinogenesis. *E. coli* bacteria is a part of normal microflora in the human gut, producing CDTs; researchers revealed their possible role in colorectal cancer progression.

Interestingly, these two toxin systems have contradictory ways and targets to cause pathogenesis in their host system. TA systems act on their cells while CDTs on the host cells; however, both aid the survival of bacteria in the host. Hence, the deep study of toxins and their molecular mechanisms can provide important details required to find their new applications in biotechnology. This, in turn, can help find new therapeutic approaches that can interfere with the harmful effects of these toxin systems during infections.

## Figures and Tables

**Table 1:** TA modules play a role in bacterial virulence

TA loci	Role of TA modules	Contribution of TA modules in virulence	Reference
MGE	Involves in the stabilisation of pathogenicity islands	Contains virulence genes or antibiotic resistance genes	(21, 24–25)
Plasmids	Plasmid maintenance	Contains virulence factors and/or antibiotic resistance factors	(13–14, 16, 33)
Chromosome	Bio-film formation	Makes the bacterial population more tolerant to antibiotics or host immune attacks	(21–22, 26, 33)

## References

[1] Levinson W (2014). Review of medical microbiology and immunology.

[2] Talaro KP, Chess B (2018). Foundations in microbiology.

[3] Tortora G, Funke B, Case C, Beauparlant (2019). Chapter 15 - Microbial mechanisms of pathogenicity. Microbiology an introduction.

[4] Wilson B, Salyers A, Whitt D, Winkler M (2011). Bacterial pathogenesis a molecular approach.

[5] Wüthrich I (2013). Bacterial protein toxin: tools to study mammalian molecular cell biology.

[6] Kedzierska B, Hayes F (2016). Emerging roles of toxin-antitoxin modules in bacterial pathogenesis. J Molecules.

[7] Cotter P, Ross R, Hill C (2013). Bacteriocins — a viable alternative to antibiotics?. Nat Rev Microbiol.

[8] Engelberg-Kulka H, Amitai S, Kolodkin-Gal I, Hazan R (2006). Bacterial programmed cell death and multicellular behavior in bacteria. PLoS Genetics.

[9] Harms A, Brodersen D, Mitarai N, Gerdes K (2018). Toxins, targets, and triggers: an overview of toxin-antitoxin biology. Mol Cell.

[10] Yamaguchi Y, Park JH, Inouye M (2011). Toxin-antitoxin systems in bacteria and archaea. Annu Rev Genet.

[11] Unterholzner SJ, Poppenberger B, Rozhon W (2013). Toxin-antitoxin systems: biology, identification, and application. Mobile Gen Elem.

[12] Gerdes K, Rasmussen P, Molin S (1986). Unique type of plasmid maintenance function: postsegregational killing of plasmid-free cells. Proceed Nat Soci Sci USA.

[13] Wen Y, Behiels E, Devreese B (2014). Toxin-antitoxin systems: their role in persistence, biofilm formation, and pathogenicity. Pathog Dis.

[14] Hayes F, Kedzierska B (2014). Regulating toxin-antitoxin expression: controlled detonation of intracellular molecular timebombs. Toxins.

[15] Gerdes K, Larsen JE, Molin S (1985). Stable inheritance of plasmid R1 requires two different loci. J Bacteriol.

[16] Bukowski M, Rojowska A, Wladyka B (2011). Prokaryotic toxin-antitoxin systems — the role in bacterial physiology and application in molecular biology. Acta Bioch Polon.

[17] Hayes F, Van Melderen L (2011). Toxins-antitoxins: diversity, evolution and function. Crit Rev Biochem Mol Biol.

[18] Yamaguchi Y, Inouye M (2011). Regulation of growth and death in *Escherichia coli* by toxin-antitoxin systems. Nat Rev Microbiol.

[19] Gerdes K, Christensen SK, Lobner-Olesen A (2005). Prokaryotic toxin antitoxin stress response loci. Nat Rev Microbiol.

[20] Marquez D, Orejas R, Portillo F (2016). Toxin-antitoxins and bacterial virulence. FEMS Microbiol Rev.

[21] Wang X, Wood T (2011). Toxin-antitoxin systems influence biofilm and persister cell formation and the general stress response. Appl Environ Microbiol.

[22] Yarmolinsky MB (1995). Programmed cell death in bacterial populations. Science.

[23] Cambray G, Guerout AM, Mazel D (2010). Integrons. Ann Rev Gen.

[24] Makarova KS, Wolf YI, Koonin EV (2009). Comprehensive comparative genomic analysis of type 2 toxin-antitoxin systems and related mobile stress response systems in prokaryotes. Biol Direct.

[25] Hernandez-Arriaga AM, Chan WT, Espinosa M, Diaz-Orezas R (2014). Conditional activation of toxin-antitoxin systems: postsegregational killing and beyond. J Microbiol Spect.

[26] Dy R, Richter C, Salmond G, Fineran P (2014). Remarkable mechanisms in microbes to resist phage infections. Ann Rev Virol.

[27] Harms A, Maisonneuve E, Gerdes K (2016). Mechanisms of bacterial persistence during stress and antibiotic exposure. Science.

[28] Hayes F (2003). Toxins-antitoxins: plasmid maintenance, programmed cell death, and cell cycle arrest. Science.

[29] Fernández-García L, Blasco L, Lopez M, Bou G, García-Contreras R, Wood T (2016). Toxin-antitoxin systems in clinical pathogens. Toxins.

[30] Sacchettini JC, Rubin EJ, Freundlich JS (2008). Drugs versus bugs: in pursuit of the persistent predator *Mycobacterium tuberculosis*. Nat Rev Microbiol.

[31] Ramage HR, Connolly LE, Cox JS (2009). Comprehensive functional analysis of *Mycobacterium tuberculosis* toxin-antitoxin systems: implications for pathogenesis, stress responses, and evolution. PLoS Gen.

[32] Keren I, Minami S, Rubin E, Lewis K (2011). Characterization and transcriptome analysis of *Mycobacterium tuberculosis* persisters. MBio.

[33] Albrethsen J, Agner J, Piersma SR, Højrup P, Pham TV, Weldingh K (2013). Proteomic profiling of *Mycobacterium tuberculosis* identifies nutrient-starvation-responsive toxin-antitoxin systems. Mol Cell Proteomics.

[34] Sala A, Bordes P, Genevaux P (2014). Multiple toxin-antitoxin systems in *Mycobacterium tuberculosis*. Toxins.

[35] Miallau L, Jain P, Arbing MA, Cascio D, Phan T, Ahn CJ (2013). Comparative proteomics identifies the cell-associated lethality of *M. tuberculosis* RelBE-like toxin-antitoxin complexes. Structure.

[36] Polom D, Boss L, Wegrzyn G, Hayes F, Kedzierska B (2013). Amino acid residues crucial for specificity of toxin-antitoxin interactions in the homologous Axe-Txe and YefM-YoeB complexes. FEBS J.

[37] Korch SB, Contreras H, Clark-Curtiss JE (2009). Three *Mycobacterium tuberculosis* Rel toxin-antitoxin modules inhibit mycobacterial growth and are expressed in infected human macrophages. J Bacteriol.

[38] Gupta M, Nayyar N, Chawla M, Sitaraman R, Bhatnagar R, Banerjee N (2016). The chromosomal parDE2 toxin-antitoxin system of *Mycobacterium tuberculosis* H37Rv: genetic and functional characterization. Front Microbiol.

[39] Tiwari P, Arora G, Singh M, Kidwai S, Narayan OP, Singh R (2015). MazF ribonucleases promote *Mycobacterium tuberculosis* drug tolerance and virulence in guinea pigs. Nat Commun.

[40] Winther K, Brodersen D, Brown A, Gerdes K (2013). VapC20 of *Mycobacterium tuberculosis* cleaves the Sarcin–Ricin loop of 23S rRNA. Nat Commun.

[41] Lee IG, Lee SJ, Chae S, Lee KY, Kim JH, Lee BJ (2015). Structural and functional studies of the *Mycobacterium tuberculosis* VapBC30 toxin-antitoxin system: implications for the design of novel antimicrobial peptides. Nucl Acids Res.

[42] Wallin G, Kamerlin SC, Aqvist J (2013). Energetics of activation of GTP hydrolysis on the ribosome. Nat Commun.

[43] Pons B, Vignard J, Mirrey J (2019). Cytolethal distending toxin subunit B: a review of structure–function relationship. Toxins.

[44] Johnson W, Lior H (1988). A new heat-labile cytolethal distending toxin (CLDT) produced by *Escherichia coli* isolates from clinical material. Microb Pathog.

[45] Gargi A, Reno M, Blanke S (2012). Bacterial toxin modulation of the eukaryotic cell cycle: are all cytolethal distending toxins created equally?. Front Cell Infect Microbiol.

[46] Pérès SY, Marchès O, Daigle F, Nougayrède JP, Herault F, Tasca C (1997). A new cytolethal distending toxin (CDT) from *Escherichia coli* producing CNF2 blocks HeLa cell division in G2/M phase. Mol Microbiol.

[47] Bezine E, Vignard J, Gladys Mirey (2014). The cytolethal distending toxin effects on mammalian cells: a DNA damage perspective. Cells.

[48] Scott DA, Kaper JB (1994). Cloning and sequencing of the genes encoding *Escherichia coli* cytolethal distending toxin. Infect Immun.

[49] Fais T, Delmas J, Serres A, Bonnet R, Dalmasso G (2016). Impact of CDT toxin on human diseases. Toxins.

[50] Elwell C, Dreyfus L (2000). DNase I homologous residues in CDTB are critical for cytolethal distending toxin-mediated cell cycle arrest. Mol Microbiol.

[51] Lara-Tejero M, Galán JE (2000). A bacterial toxin that controls cell cycle progression as a deoxyribonuclease I-like protein. Science.

[52] Guerra L, Cortes-Bratti X, Guidi R, Frisan T (2011). The biology of the cytolethal distending toxins. Toxins.

[53] Scuron M, Boesze-Battaglia K, Dlakic M, Shenker B (2016). The cytolethal distending toxin contributes to microbial virulence and disease pathogenesis by acting as a tri-perditious toxin. Front Cell Infect Microbiol.

[54] Nesic D, Hsu, Stebbins CE (2004). Assembly and function of a bacterial genotoxin. Nature.

[55] Lara-Tejero M, Galán JE (2001). CDTA, CDTB and CDTC form a tripartite complex that is required for cytolethal distending toxin activity. Infect Immun.

[56] McSweeney L, Dreyfus L (2004). Nuclear localization of the *Escherichia coli* cytolethal distending toxin CdtB subunit. Cell Microbiol.

[57] Nishikubo S, Ohara M, Ueno Y, Ikura M, Kurihara H, Komatsuzawa H (2003). An N-terminal segment of the active component of the bacterial genotoxin cytolethal distending toxin B (CDTB) directs CDTB into the nucleus. J Biol Chem.

[58] Fahrer J, Huelsenbeck J, Jaurich H, Dörsam B, Frisan T, Eich M (2014). Cytolethal distending toxin (CDT) is a radiomimetic agent and induces persistent levels of DNA double-strand breaks in human fibroblasts. DNA Rep.

[59] Niida H, Nakanishi M (2006). DNA damage checkpoints in mammals. Mutagenesis.

[60] Guidi R, Guerra L, Levi L, Stenerlöw B, Fox JG, Josenhans C (2013). Chronic exposure to the cytolethal distending toxins of Gram-negative bacteria promotes genomic instability and altered DNA damage response. Cell Microbiol.

[61] Shenker B, Dlakic M, Walker L, Besack D, Jaffe E, LaBelle E (2007). A novel mode of action for a microbial-derived immunotoxin: the cytolethal distending toxin subunit B exhibits phosphatidylinositol 3,4,5-triphosphate phosphatase activity. J Immunol.

[62] Seminario M, Wange R (2003). Lipid phosphatases in the regulation of T cell activation: living up to their PTEN-tial. Immunol Rev.

[63] March M, Ravichandran K (2002). Regulation of the immune response by SHIP. Seminars in Immunology.

[64] Dlakic M (2001). Is CdtB a nuclease or a phosphatase?. Science.

[65] Jinadasa R, Bloom S, Weiss R, Duhamel G (2011). Cytolethal distending toxin: a conserved bacterial genotoxin that blocks cell cycle progression, leading to apoptosis of a broad range of mammalian cell lineages. Microbiol.

[66] Suguimoto E, Silva M, Kawamoto D, Chen C, DiRienzo J, Mayer M (2014). The cytolethal distending toxin of *Aggregatibacter actinomycetemcomitans* inhibits macrophage phagocytosis and subverts cytokine production. Cytokine.

[67] Shenker B, Ojcius D, Walker L, Zekavat A, Scuron M, Boesze-Battaglia K (2015). *Aggregatibacter actinomycetemcomitans* cytolethal distending toxin activates the NLRP3 inflammasome in human macrophages, leading to the release of proinflammatory cytokines. Infect Immun.

[68] Svensson LA, Tarkowski A, Thelestam M, Lagergard T (2001). The impact of *Haemophilus ducreyi* cytolethal distending toxin on cells involved in immune response. Microb Pathog.

[69] Pickett C, Lee R, Eyigor A, Elitzur B, Fox E, Strockbine N (2004). Patterns of variations in *Escherichia coli* strains that produce cytolethal distending toxin. Infect Immun.

[70] Hinenoya A, Naigita A, Ninomiya K, Asakura M, Shima K, Seto K (2009). Prevalence and characteristics of cytolethal distending toxin-producing *Escherichia coli* from children with diarrhea in Japan. Microbiol Immunol.

[71] Graillot V, Dormoy I, Dupuy J, Shay JW, Huc L, Mirey G, Vignard J (2016). Genotoxicity of cytolethal distending toxin (CDT) on isogenic human colorectal cell lines: potential promoting effects for colorectal carcinogenesis. Front Cell Infect Microbiol.

[72] He Z, Gharaibeh R, Newsome R, Pope J, Dougherty M, Tomkovich S (2018). *Campylobacter jejuni* promotes colorectal tumorigenesis through the action of cytolethal distending toxin. BMJ J.

[73] Fearon ER, Vogelstein B (1990). A genetic model for colorectal tumorigenesis. Cell.

[74] Narayan S, Sharma R (2015). Molecular mechanism of adenomatous polyposis coli-induced blockade of base excision repair pathway in colorectal carcinogenesis. Life Sci.

[75] Koyokawa E, Minato H (2014). Activated K-RAS and its effect on morphological appearance. J Biochem.

[76] Bonnet M, Buc E, Sauvanet P, Darcha C, Damien D, Bruno P (2014). Colonization of the human gut by *E. coli* and colorectal cancer risk. Human Cancer Biol.

[77] Buc E, Dubois D, Sauvanet P, Raisch J, Delmas J, Darfeuille-Michaud A (2013). High prevalence of mucosa-associated *E. coli* producing cyclomodulin and genotoxin in colon cancer. PLoS ONE.

[78] Bezine E, Malaisé Y, Loeuillet A, Chevalier M, Boutet-Robinet E, Salles B (2016). Cell resistance to the cytolethal distending toxin involves an association of DNA repair mechanisms. Sci Rep.

